# ReSurfEMG: A Python Package for Comprehensive Analysis of Respiratory Surface EMG

**DOI:** 10.3390/s25206465

**Published:** 2025-10-19

**Authors:** Robertus Simon Petrus Warnaar, Candace Makeda Moore, Walter Baccinelli, Farnaz Soleimani, Dirk Wilhelm Donker, Eline Oppersma

**Affiliations:** 1 Cardiovascular and Respiratory Physiology, Technical Medical Centre, University of Twente, 7500 AE Enschede, The Netherlands; f.soleimani@utwente.nl (F.S.); e.mos-oppersma@utwente.nl (E.O.); 2 Netherlands eScience Center, 1098 XH Amsterdam, The Netherlands; 3 Intensive Care Centre, University Medical Centre Utrecht, 3508 GA Utrecht, The Netherlands

**Keywords:** respiratory surface electromyography, software, Python, open-source, signal processing, quality assessment

## Abstract

**Highlights:**

**What are the main findings?**

- ReSurfEMG is a comprehensive Python package for advanced respiratory sEMG analysis.
- Signal processing methodology and settings profoundly affect sEMG signal quality.

**What is the implication of the main finding?**

- ReSurfEMG facilitates comprehensive reporting as a citable methodological reference.
- ReSurfEMG lays the open-source groundwork for methodological standardization.

**Abstract:**

In patients with respiratory failure, mechanical ventilation aims to balance respiratory muscle loading and gas exchange. The interplay between the ventilator and the respiratory muscles is an increasingly recognized factor in tailoring ventilatory support. Surface electromyography (sEMG) offers a non-invasive modality to monitor the respiratory muscles. The sEMG signal, however, requires elaborate processing, which is limitedly standardized and documented. This paper presents the Respiratory Surface Electromyography (ReSurfEMG) package, an open-source Python package for respiratory sEMG analysis developed to address these challenges. ReSurfEMG integrates denoising, feature extraction, and quality assessment in one dedicated library. The effects of over- and under-filtering were compared to ReSurfEMG default settings regarding waveform duration, time-to-peak, amplitude, electrical time product (ETP), pseudo-slope, pseudo-signal-to-noise ratio (SNR), area under the baseline (AUB), and bell-curve error. Under-filtering increased amplitudes (+21%) and ETPs (+10%). Over-filtering smoothed sEMG waveforms, reducing amplitude (−58%), ETP (−39%), and pseudo-slope (−49%), while waveform duration and time-to-peak increased. Default ReSurfEMG settings provided the highest SNRs with similar or lower AUBs and bell-curve errors. The ReSurfEMG library integrates advanced methods dedicated to respiratory sEMG analysis. Systematic assessment using ReSurfEMG showed that signal processing settings affect sEMG features. ReSurfEMG enables reproducible signal processing, facilitating the standardization of respiratory sEMG analysis.

## 1. Introduction

Many acute and chronic medical conditions may at some point culminate in severe respiratory failure requiring mechanical ventilation. The latter can temporarily be provided in an Intensive Care Unit (ICU) setting or ambulatory as long-term home mechanical ventilation. All mechanical ventilatory support strategies share the common goal of supporting or replacing the function of the respiratory muscles. These muscles form the human respiratory pump, which generates continuous breathing effort throughout the day, with the diaphragm as its primary muscle [1]. During respiratory failure, work of breathing increases or the force-generating capacity of the respiratory muscles decreases, such that the diaphragm and accessory respiratory muscles are unable to ensure adequate ventilation and oxygenation. Mechanical ventilation aims to improve gas exchange and relieve the patient’s work of breathing. However, inadequately tailored ventilatory support can cause over-assistance, under-assistance, or patient-ventilator asynchrony and lead to patient discomfort, ventilator intolerance, and ultimately poor patient outcomes [2,3,4,5]. The importance of monitoring respiratory muscle activity is therefore increasingly recognized as an adjunct to optimally inform tailoring of ventilatory support.

Respiratory muscle activation is initiated by the muscle fiber’s electrical depolarization, which is measurable at the bedside using surface electromyography (sEMG) [6]. Respiratory sEMG offers an accessible modality, basically as easy in operation as the common electrocardiogram (ECG). This ease of use may support widespread respiratory muscle monitoring in a variety of care environments, from the ICU to home mechanical ventilation [7,8,9]. However, the inherent cardiac and adjacent muscle crosstalk in respiratory sEMG signals [10] necessitates elaborate signal processing. Moreover, methodologies vary widely in the literature and are generally not discussed in detail. These aspects complicate interpretation and comparison of study findings, hampering the translation of the respiratory sEMG technology from basic research towards widespread use in clinical practice.

Open-source software for sEMG signal analysis has great potential to improve methodological transparency while assuring data quality and comparability. However, existing open-source software for general EMG signal processing and analysis only partially supports the specific needs of respiratory sEMG applications (Table 1) [11,12,13]. These packages often focus on general neuromuscular or multi-modal physiological signal analysis and are therefore not tailored to the specific requirements of respiratory sEMG. As a result, research groups often develop custom signal analysis pipelines, leading to methodological variability and limited reproducibility [14,15,16]. The ReSurfEMG project was initiated to address this gap by integrating all essential steps, from raw signal processing to feature extraction, into a single dedicated library [17]. ReSurfEMG focuses on single-channel respiratory sEMG analysis, integrating ECG artifact removal, envelope-based feature extraction, and signal quality assessment into a unified pipeline tailored for analysis of respiratory drive, effort, and timing. This paper introduces the ReSurfEMG package and illustrates how variations in signal processing can substantially affect the resulting features, highlighting the importance of consistent and well-documented analysis practices to ensure reproducibility and interpretability in respiratory sEMG research.

## 2. Software Description

### 2.1. The ReSurfEMG Python Package

**Figure 1:**
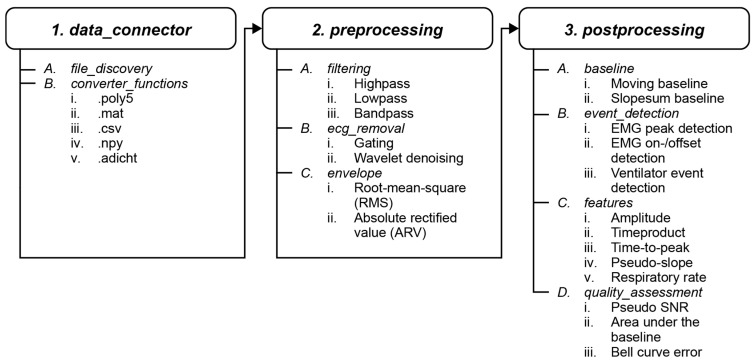
ReSurfEMG module workflow for respiratory sEMG processing.

The ReSurfEMG Python^®^ (Python Software Foundation, Wilmington, DE, USA) package forms the core of the project, providing specialized methods for respiratory sEMG analysis. The package offers standardized methods for data reading, pre-processing, post-processing, and data visualization (Table 2, Figure 1). ReSurfEMG is developed and tested for Python 3.9–3.12 running on Linux, Windows, and OSx, and distributed via the Python Package Index (PyPI) (https://pypi.org/project/resurfemg/ accessed on 29 September 2025) under the Apache 2.0 license. Documentation is hosted on Read the Docs (https://resurfemg-org.github.io/ReSurfEMG/ accessed on 29 September 2025). Within the ReSurfEMG/ReSurfEMG (https://github.com/resurfemg-org/ReSurfEMG accessed on 29 September 2025) repository, the directory *ReSurfEMG* contains the core package.

**Figure 2:**
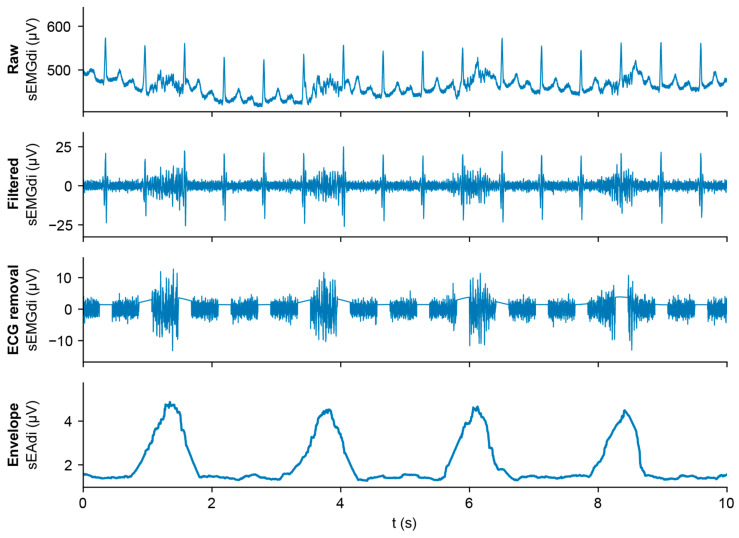
sEMG processing pipeline. From top to bottom: raw signal, band-pass filtered signal, ECG removed by gating, calculated envelope.

The package is organized in subpackages according to the fundamental steps in sEMG signal analysis. After loading data or generating simulated data using the *data_connector* modules, the *preprocessing* subpackage offers a three-step method for denoising the signal, including filtering, ECG removal, and envelope calculation (Figure 2). During *post_processing*, the envelope *baseline* is determined, which is needed for *event_detection*, *feature* calculation, and eventually sEMG *quality_assessment*. These subpackages will be described in more detail in the paragraphs below.

#### 2.1.1. Data Connector: Reading sEMG Data

The *converter_functions* module of the ReSurfEMG *data_connector* subpackage is able to read a variety of formats as acquired by commonly used biomedical amplifiers, i.e., .poly5, .adicht, .mat, .csv, and .npy, thereby facilitating interoperability and reusability. Loaded data is transformed to the standardized time series format as used throughout the package: a NumPy *NxM* array with *N* data channels and *M* samples. Additional modules in this subpackage are included for automated discovery of relevant files and directories (*config*, *file_discovery*), for generating synthetic data for testing and benchmarking (*synthetic_data*), and for reading data in the .Poly5 (*tmsisdk_lite*) and .adicht (*adicht_reader*) formats.

The library provides a standardized framework for object-oriented handling and processing of data through its *data_classes*. Data is stored in *TimeSeries* class objects, which are grouped in a *TimeSeriesGroup* class object. These classes have embedded ReSurfEMG methods to transform the raw data into an envelope signal and calculate the features of interest. As a result, a comprehensive signal analysis pipeline can be realized with just a few lines of code, whereas full flexibility is maintained to tailor the underlying methods to specific applications.

#### 2.1.2. Pre-Processing: Filtering

Raw sEMG recordings are highly contaminated by baseline wander, movement artifacts, and the ECG, concealing the respiratory muscle activity. The *preprocessing* subpackage provides the functionality to denoise the raw sEMG data and transform it into an envelope: the tracing of respiratory muscle activity.

The first step in denoising is filtering baseline wander, movement artifacts, and powerline noise. Low, high, notch, and bandpass butter filters are implemented in the *filtering* module. The sEMG frequency content typically ranges between 10 and 500 Hz [18], showing most frequency power between approximately 20 and 150 Hz [19]. The main power of baseline wander occurs in the very low range below 0.5 Hz [20], whereas movement artifacts are usually in the 0–20 Hz frequency range [18]. These artifacts can be eliminated by high-pass filtering with a cutoff around 10–20 Hz without significantly affecting the EMG frequency bands. Yet, such filtering distorts the ECG waveform [21], which complicates QRS complex detection in subsequent steps. The method for QRS complex detection (*detect_ecg_peaks*) is therefore designed for unfiltered ECG/EMG recordings and might perform suboptimally when applied to filtered data.

Powerline noise is reflected in the midrange of high-power frequencies of the sEMG with a center frequency of 50 Hz or 60 Hz. This interference is ideally prevented during recording, e.g., by using shielded cables and minimizing skin-electrode impedance. Powerline interference can be eliminated by applying a notch filter around the powerline center frequency. However, such filtering destroys significant signal information and could introduce substantial phase shift in the neighboring frequency bands [18]. As limited sEMG power is expected above 500 Hz [19], signals are typically low-pass filtered at 500 Hz. When lower sampling rates are used, the cutoff frequency is adjusted accordingly to remain below the Nyquist limit [18].

#### 2.1.3. Pre-Processing: ECG Removal

Removal of ECG artifacts is challenging because the ECG amplitude substantially exceeds that of the sEMG, while its frequency spectrum has significant overlap with the sEMG [21]. The *ecg_removal* module contains two methods for ECG removal—gating and wavelet denoising [22]—as well as supporting methods for QRS complex detection.

**Figure 3:**
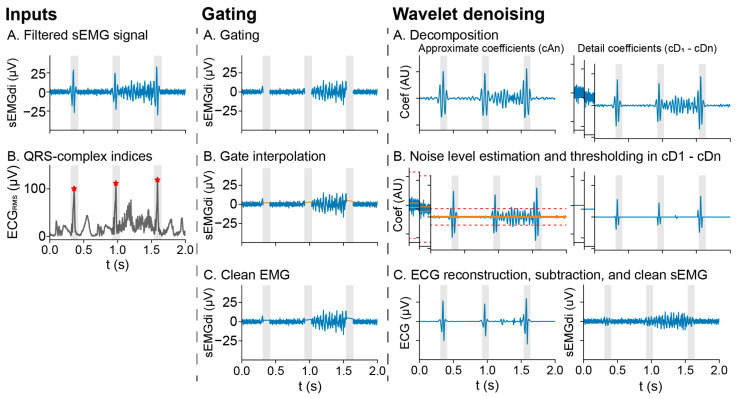
ECG removal methods—Gating: A. Eliminate data within gate windows. B. Fill the removed segments using the selected method. Wavelet denoising: A. Decompose the signal using the chosen wavelet. B. Estimate the noise level using the median absolute deviation (MAD, orange), scale it by dividing by 0.6745, and multiply by the *fixed_threshold* to determine the noise threshold (red). Suppress noise by thresholding coefficients below the noise threshold. C. Reconstruct the denoised ECG from the thresholded coefficients and subtract it from the sEMGdi to obtain the clean sEMGdi.

Gating eliminates QRS peaks by eliminating the data in the windows around the QRS complexes (Figure 3). The gate width and filling methods are the main determinants of the gating result, as the gates should cover the entire QRS artifact, and the fill should be representative of the eliminated EMG signal. Four methods are available to fill the gates: (1) zeroes, (2) the average of the prior segment or, if non-existent, the subsequent segment, and interpolation samples before and after the gate, and either (3) the raw or (4) root mean square (RMS) envelope of the signal. 

Wavelet denoising uses the stationary wavelet transform (SWT) to estimate a noise-free ECG, which is then subtracted from the original signal to obtain the denoised sEMG. The SWT breaks down the signal into multiple frequency bands (Figure 3), determined by the decomposition level *n*. The lowest frequency band, called the approximate coefficients, extends up to the cutoff frequency *fc* = *fs*∕2*^n^* for a given sampling frequency *fs*. The detail coefficients represent the higher frequency components, where each decomposition level covers one octave up to *fs*. The approximate coefficients are assumed to contain only non-EMG activity and are all included in the noise-free ECG. For the detail coefficients, a noise threshold is set per decomposition level, which is calculated as the running median absolute deviation (MAD) divided by 0.6745, multiplied by the *fixed_threshold*. Coefficients below this noise threshold are considered noise and eliminated from the noise-free ECG. Increasing the *fixed_threshold* reduces the number of coefficients included in the cleaned ECG, preserving more signal power for the denoised EMG.

#### 2.1.4. Pre-Processing: Envelope

As the EMG signal is stochastic in nature, a signal envelope is needed to obtain a meaningful estimate of the muscle’s surface electrical activity over time and identify separate breaths from the continuous signal. Specifically for the diaphragm, the envelope is referred to as the surface electrical activity of the diaphragm (sEAdi). Importantly, the electrical activity of other respiratory muscles could be processed in a similar manner.

ReSurfEMG includes implementations of both the running root-mean-square (RMS) and average rectified value (ARV) for calculating envelopes [23]. RMS squares the signal before averaging, whereas ARV only rectifies the signal. As a result, RMS yields higher amplitudes but is also more sensitive to high-amplitude outliers. The key parameter for both the ARV and RMS methods is the *window_length*. The larger the window length, the more precise the estimated sEAdi amplitude [23]. This increase in precision comes at the cost of a decreased time resolution, as the signal is smoothed to a greater extent. To signify the uncertainty in envelope estimation, ReSurfEMG provides functions to estimate the confidence intervals for RMS and ARV based on the central limit theorem (*rolling_rms_ci*, *rolling_arv_ci*).

#### 2.1.5. Postprocessing: Event Detection

The *postprocessing* subpackage includes functions to detect breathing events, extract features, and assess the quality of the sEAdi signal on a breath-by-breath basis. Its *baseline* module supports event detection by offering various methods to calculate moving baselines from the envelope. Both a percentile-based method [24] and a slope sum baseline [25] are implemented. The slope sum baseline augments the sEAdi signal with its smoothed derivative to better capture rapid respiratory changes and calculates a moving baseline over this augmented signal. The main parameters of these methods are the window length for smoothing the baseline, the step size for balancing granularity against calculation speed, and, for the slope sum baseline, the augmented percentage, which is the fraction of the envelope derivative added to the regular moving baseline.

The *event_detection* module identifies the waveform maximum (*peak_idx*) of each breath, as well as its on- (*start_idx*) and offsets (*end_idx*). On- and offsets are determined either by baseline crossing of sEAdi (*onoffpeak_baseline_crossing*) or by extrapolating its maximum slopes to the baseline level (*onoffpeak_slope_extrapolation*). These functions provide feedback on the on- and offset detection validity, identifying where onsets occur after the **waveform** maximum, offsets occur before the maximum, and **waveform** windows overlap, i.e., the onset or offset of one waveform overlaps with the window of another. The *event_detection* module, moreover, includes functions for identifying ventilator-supported breaths and occluded breaths. It also allows for matching **waveforms** from various tracings based on the peak timings (*find_linked_peaks*).

#### 2.1.6. Postprocessing: Features

**Figure 4:**
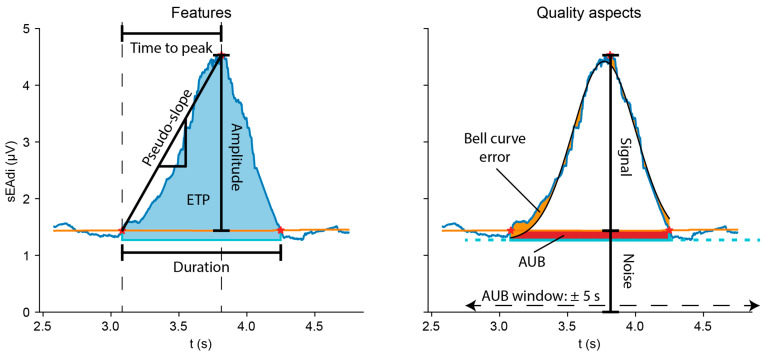
sEAdi waveform features (**left**) and quality aspects (**right**). Pseudo SNR = Signal/Noise. AUB is calculated between the moving baseline and the sEAdi minimum in the AUB window. AUB (red) and bell errors (orange) are expressed as a percentage of the ETP. Abbreviations ETP Electrical time product, AUB Area under the baseline.

The *features* module has implementations to calculate the sEAdi amplitude and electrical time product (ETP, i.e., the area under the curve) as measures of magnitude of respiratory muscle activity (Figure 4). The time to peak (both absolute and relative to the breath length), respiratory rate, and pseudo-slope are implemented as measures of activation rate and frequency.

#### 2.1.7. Postprocessing: Quality Assessment

The ReSurfEMG library automates quality assessment of the sEAdi waveforms and parameters, flagging low-quality waveforms based on predefined criteria [25,26]. Quality criteria were introduced to identify non-physiological waveforms, which reduce the variability in sEMG-based measures [25]. Waveform quality is evaluated by comparing the signal of interest to the noise level and the baseline variability. The library approximates the signal-to-noise ratio (SNR) based on the peak amplitude relative to the moving baseline (*pseudo_snr*) and quantifies baseline-related uncertainty in the ETP using the area under the baseline (AUB, Figure 4). AUB reflects how variability at the baseline level contributes to uncertainty in the ETP by expressing this variability relative to the total waveform area. The *percentage_under_baseline* method calculates this uncertainty by dividing the AUB over the total ETP of the waveform. The *detect_local_high_aub* method identifies locally high noise levels, while the *detect_extreme_timeproducts* method identifies waveforms with extraordinarily high ETPs, which both arise due to motion and crosstalk artifacts.

The waveform morphology is assessed by comparing the sEAdi waveform to a bell shape, representing the physiological pattern of respiratory EMG waveforms [27]. Deviations from this morphology, as evaluated by the *evaluate_bell_curve_*error method, indicate the remainder of artifacts, thereby rendering such a waveform not representative nor suitable for further analysis.

Quality indicators of the overall recording and detected peaks are the agreement of EMG waveforms with signals, e.g., flow, volume, pressure, or ECG. The *interpeak_dist* and *evaluate_respiratory_rates* methods compare the number of EMG peaks to the ECG-based heart rate and pneumatic respiratory rate. The *evaluate_event_timing* function verifies if the peak timings in matched peak sets fall within a specified window, whereas the *detect_non_consecutive_manoeuvres* checks if maneuver-based efforts are temporally separated by regular breaths, ensuring that only non-consecutive maneuvers are included for analysis.

### 2.2. The ReSurfEMG Repository

The core Python package is accompanied by a researcher and a dashboard interface. The researcher interface includes a step-by-step “Getting Started” guide (available at https://github.com/resurfemg-org/ReSurfEMG#getting-started accessed on 29 September 2025) for new users, along with example Jupyter notebook workflows (available at https://github.com/resurfemg-org/ReSurfEMG/notebooks accessed on 29 September 2025) for off-the-shelf analysis of respiratory sEMG and the development of new features. These notebooks are accessible to users with limited coding experience, whereas they offer great flexibility in the development of dedicated data analysis pipelines. The dashboard interface (available at https://github.com/resurfemg-org/ReSurfEMG-dashboard accessed on 29 September 2025), on the other hand, provides a zero-code way to perform standardized sEMG analyses, thereby facilitating clinical researchers in the fast translation of newly developed advanced signal analysis methodology into clinically relevant results.

## 3. Materials and Methods

### 3.1. Experimental Setup

All analyses were performed using Python 3.12 using the developer (*dev*) installation of ReSurfEMG version 1.0.2 [28]. All Jupyter Notebooks to reproduce the analyses are available at (Available at: https://github.com/resurfemg-org/ReSurfEMG/tree/main/notebooks/researcher_interface/software_paper accessed on 29 September 2025). The effect of processing settings on the resulting signals and parameters is evaluated by analyzing patient data of a previously published study on sEAdi at varying ventilator settings [25]. The sEMG was measured on the right side over the eighth intercostal space in the anterior axillary line with pre-gelled Ag/AgCl electrodes (3M™ Red Dot™ 2560 electrodes, 3M Deutschland GmbH, Neuss, Germany). The electrodes were connected via actively shielded bipolar electrode cables (TMSi, Oldenzaal, The Netherlands) to a Mobi-6 device (TMSi, Oldenzaal, The Netherlands, 12.2 nV/bit, amplification factor: 19.5). The skin was cleansed with alcohol before electrode application. EMG signals were acquired at a sample rate of 2048 Hz using the 2016 TMSi MATLAB interface. The raw signals were loaded directly into ReSurfEMG using the *data_connector* module without any preprocessing beyond analog-to-digital conversion with the specified gain. For this paper, sEMG data of the diaphragm is used as an example, but it should be noted that sEMG data of other respiratory muscles could be processed in a similar manner.

### 3.2. Data Analysis

Filtering, ECG removal, envelope, and baseline calculation were performed as specified in Table 3. The function settings were varied in specified ranges, maintaining the other settings on the ReSurfEMG default values, as derived from the EMG literature. Ranges were chosen to capture the effects of over- and under-filtering, defined, respectively, as applying more or less aggressive filtering and smoothing than recommended by respiratory sEMG guidelines and literature (Section 2.1). The analysis is concluded by analyzing one dataset with the default, over- and under-filtering settings, comparing the difference in sEAdi waveform duration, time-to-peak, amplitude, ETP, pseudo-slope, pseudo-SNR, AUB, and bell-curve error.

## 4. Results

### 4.1. Filtering

**Figure 5:**
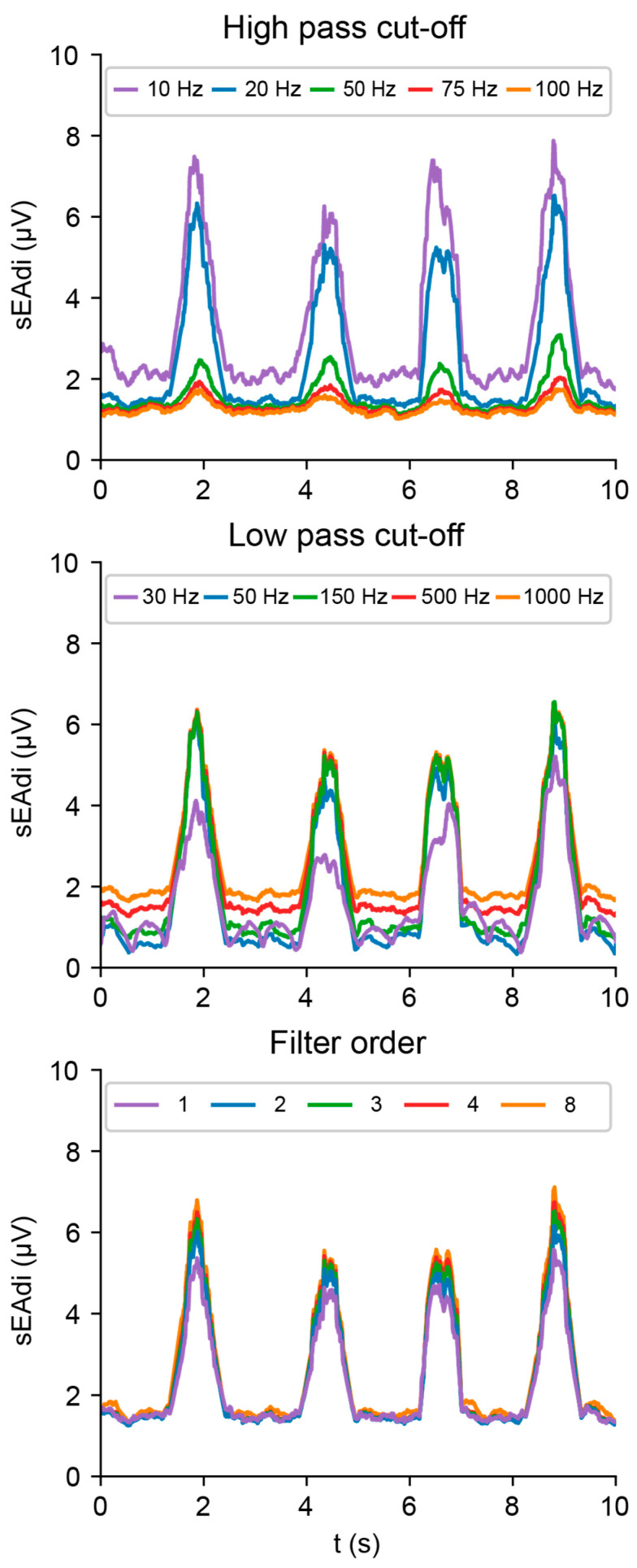
Effect of the high-pass cutoff (**top**) and low-pass cutoff frequencies (**middle**) and filter order (**bottom**) on the signal envelope.

The EMG envelope was primarily affected by the high-pass cutoff frequency (*cf_hp_*), as compared to the low-pass cutoff frequency (*cf_lp_*) and filter order (Figure 5). For *cf_hp_* below 20 Hz, envelope irregularity increased, as the frequency power of sEMG concentrated between 20 and 150 Hz [19]. Including frequency bands below 20 Hz mainly adds slow, non-EMG activity. For *cf_hp_* above *20 Hz,* frequency bands with considerable sEMGdi power are eliminated, as shown by the decrease in area under the curve and peak amplitude.

*cf_lp_* settings below 150 Hz add irregularity to the baseline and waveforms, as the reduced signal bandwidth is associated with less robust amplitude estimation [23]. In this dataset, *cf_lp_* settings above 150 Hz primarily shift the entire signal envelope upwards, indicating limited additional EMG signal power in this frequency range.

Higher filter orders create sharper transitions between the pass- and stopband in the frequency domain, capturing more frequency power in the desired ranges. Consequently, higher filter orders increase the envelope amplitudes. However, increasing the filter order beyond 3 had minimal effect.

### 4.2. ECG Removal

**Figure 6:**
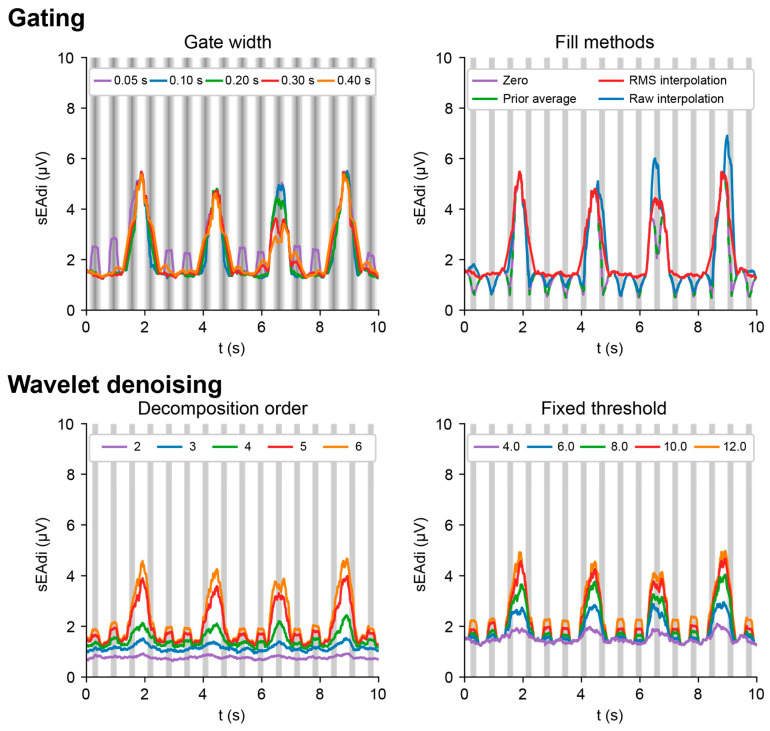
Effects of gating (**top**) and wavelet denoising (**bottom**) on ECG removal. Gate width (**left**) and gate filling methods (**right**) are varied for gating. The decomposition order (**left**) and fixed threshold (**right**) are varied for wavelet denoising.

Figure 6 illustrates the effects of the gating and wavelet denoising settings on sEAdi. For gating windows shorter than 200 ms, envelope spikes arose in the QRS windows, with the ECG peak amplitudes nearing the level of respiratory muscle activity. The default gate width is set to 200 ms, considering the healthy QRS complex duration is up to 110 ms, though its morphology varies by lead [29]. A gate width of 200 ms provides a safety margin around this 110 ms, especially for the application in an ICU where cardiac conduction disorders are common [30].

The RMS interpolation provided the smoothest envelopes, as compared to filling with zeros, the prior average, or interpolation of raw data. The prior average and zeros method are virtually identical and drop to near-zero values halfway through the gating window, as expected based on the zero-mean of the sEMG signal [31]. The raw interpolation deviated locally from the prior average and zeros methods due to the coincidental interpolation between high-amplitude sEMG waveforms. Consequently, the filling by prior average and raw interpolation has little application in gating raw EMG data but could allow for gating envelope signals.

For wavelet denoising, the envelope amplitude increased with higher decomposition levels and higher fixed thresholds, although residual ECG activity increased accordingly. The envelope amplitudes at a *fixed_threshold* = 12.0 and *n* = 5 were similar to those obtained from gating. The resulting noise threshold of 12.0 σ is considerably higher than the previously proposed 4.5 σ [22]. As wavelet denoising performed better on the repository test data (see Supplementary Materials File S1), this difference in thresholds is likely due to the spectral content of the respiratory sEMG. The test data was generated by modulating white noise (*data_connector.synthetic_data* and *data_pipelines.synthetic_data* methods), which therefore had a broader power spectrum compared to patient data. The preferred method for ECG removal thus depends on signal characteristics, such as signal-to-noise ratio and frequency content, as well as the outcomes of interest, i.e., respiratory rate, amplitude, area under the curve, or peak timing.

### 4.3. Envelope

**Figure 7:**
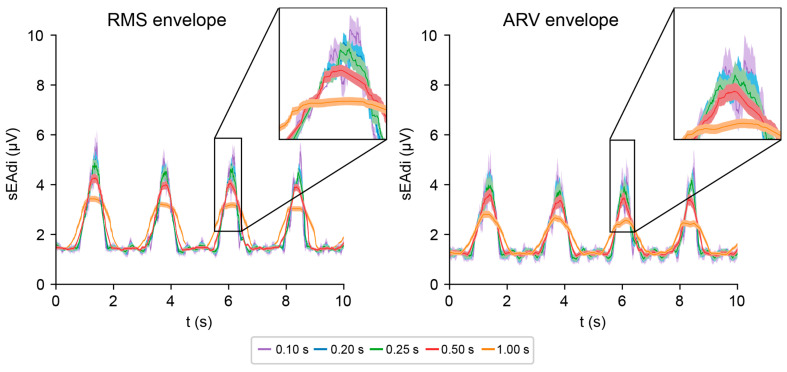
Effects of window length on RMS (**left**) and ARV (**right**) envelopes.

The effects of window length are similar for the ARV and RMS envelopes (Figure 7). The longer the window length, the smoother the envelope and the smaller the error margins. At the same time, smoother envelopes result in temporal blurring of waveforms, affecting the timing of waveform on- and offsets and flattening the waveforms. The envelope amplitudes are higher for RMS than for ARV due to the squaring before averaging, which emphasizes the high-amplitude datapoints.

### 4.4. Moving Baseline and Event Detection

**Figure 8:**
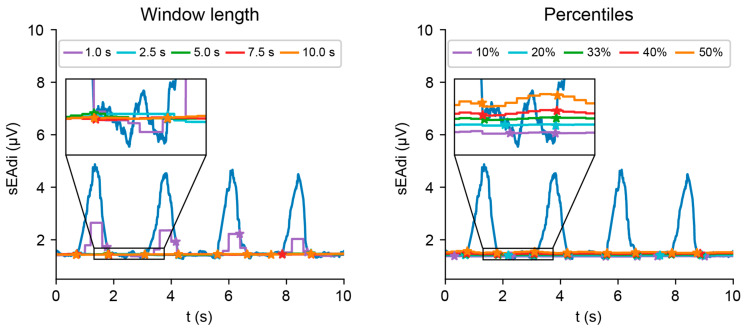
Effect of baseline window length (**left**) and percentile (**right**) on the detection of breath on- and offset.

The moving baselines exhibit highly similar macroscopic behavior with varying window lengths and percentiles (Figure 8). Only the baseline with a 1 s window length deviates from this behavior by following the waveform morphology, indicating a too-short window length. Upon closer inspection, the baseline shifts upwards according to the baseline percentiles. When the waveform continues to rise or fall beyond all baselines (Figure 8, zoom box, right waveform), the shift in on- and offset detection is already up to 200 ms from the 10th to 50th percentile. Even larger shifts are observed for waveforms with more sEAdi irregularity (Figure 8, right zoom box, left waveform). These shifts consequently impact key sEAdi features, such as amplitude, area under the curve, and peak timing.

### 4.5. Features and Quality Assessment

**Figure 9:**
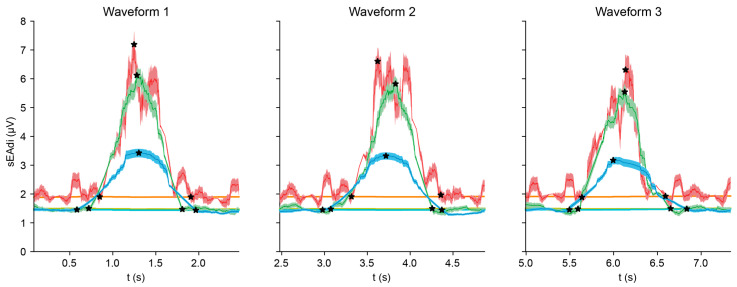
Effects of under- (red) and over-filtering (blue) as compared to the default ReSurfEMG settings (green).

Figure 9 and Table 4 compare ReSurfEMG default settings with over- and under-filtering. The default and under-filtering were similar in terms of amplitude and ETP, with under-filtering showing a 21% higher amplitude and 10% higher ETP as compared to the default. Over-filtering reduced amplitude by 58% and ETP by 39%. The default settings provided the best signal-to-noise ratios and lower or similar AUBs and bell errors. Although over-filtering reduced the bell error by 42%, it heavily reduced the signal amplitude and ETP, while waveform duration increased by 18%.

## 5. Discussion

This paper presents ReSurfEMG, an open-source Python package designed for advanced, comprehensive, and understandable analysis of respiratory sEMG data. Systematic assessment of clinical data illustrates that sEMG features and signal quality aspects importantly depend on the applied pre- and post-processing. These findings highlight the importance of detailed documentation of analysis pipelines to ensure that results are interpretable, comparable, and reproducible. ReSurfEMG addresses this need by providing a transparent framework for respiratory sEMG analysis. Its clear processing workflows support consistent comparison of findings across studies, advancing the respiratory sEMG methodology towards clinical application.

### 5.1. Respiratory sEMG: From Research to the Bedside

The analysis of respiratory sEMG has been considered to be technically demanding, as reflected by the wide range of proposed signal analysis approaches [10,22]. The current study demonstrates that signal processing methodology profoundly affects the resulting sEMG signal, stressing the need for standardization to improve data quality and comparability of individual patient measurements and clinical scientific studies. However, standardization should not imply rigid uniformity. Each clinical question brings its own parameters of interest, which in turn guide analytical requirements, such as amplitude accuracy or temporal resolution. The analytical workflows are further shaped by the relevant clinical contexts. For instance, ICU patients may exhibit altered ECG morphology [30], while patients with chronic obstructive pulmonary disease (COPD) often show abdominal muscle crosstalk [32]. These variations may require tailored signal processing strategies. Standardization efforts should aim for consistency within specific contexts of use while still allowing for methodological differentiation across applications and clinical domains.

To enable domain-specific standardization, methods should be reported transparently with clear substantiation of any deviations from common practices. Such transparency enables researchers to identify common practices and evaluate their suitability for specific clinical or research contexts. This process fosters consensus on best practices and lays the groundwork for evidence-based standardization, ultimately improving the consistency and comparability of study results.

The ReSurfEMG library facilitates both transparent reporting and standardization. Signal analysis workflows can be concisely reported by citing the library, as demonstrated by a recent clinical study investigating sEAdi in ICU patients [25,33]. The groundwork for community-driven developments of the ReSurfEMG package has also been laid with the initial version of the software reviewed and published in the Journal for Open Source Software [34]. ReSurfEMG is provided fully open-source, grounded in physiological sEMG literature, and extensively documented; the library provides a strong foundation for future standardization efforts.

### 5.2. Limitations

This study is based on data from an individual patient, acquired using a specific acquisition setup, i.e., over the eighth intercostal space in the anterior axillary line [25]. sEMG amplitude and frequency content are, however, affected by both the acquisition setup and the individual anatomical and physiological patient characteristics [35]. Although exact numerical outcomes of the performed analyses may vary due to these subject- and setup-related factors, the results primarily serve to illustrate that signal processing choices substantially influence sEMG-derived metrics. This underscores the necessity for transparent and reproducible workflows, as enabled by ReSurfEMG. Quantitative benchmarking across diverse datasets would be a logical next step but requires a sufficiently large and varied cohort to account for inter-subject and setup-related variability.

### 5.3. Future Directions

While the ReSurfEMG package currently encompasses all phases of respiratory sEMG processing, ongoing development is essential to accelerate the translation of technical innovations into clinical research. Novel methodologies are typically implemented in proprietary software [22,36,37,38], which limits their accessibility. Incorporation of these advanced techniques into the open-source ReSurfEMG package and the easy-to-use dashboard will enhance the accessibility and expedite adoption of state-of-the-art signal analysis methods.

In parallel, future development will focus on advancing ReSurfEMG’s clinical applicability. The package enables breath-by-breath quantification of respiratory drive and effort, which may inform the titration of ventilator support in ICU patients. These capabilities have already been applied in exploratory clinical studies [25,26]. Future work will focus on large-scale validation across diverse patient populations and ventilator settings. Additionally, ReSurfEMG provides the analytical foundation for future detection of patient-ventilator asynchrony, a key contributor to diaphragm injury and ventilator intolerance [2,3,4,5]. While current analyses are performed offline, ReSurfEMG will be developed toward real-time monitoring, supporting dynamic bedside decision-making to optimize ventilator settings. Ultimately, this will advance the respiratory sEMG methodology towards a widespread clinical application.

## 6. Conclusions

The open-source Python library ReSurfEMG offers advanced and comprehensive methods for processing and analysis of respiratory sEMG data integrated in one dedicated package. Through systematic evaluation using ReSurfEMG, signal processing settings were shown to profoundly affect sEMG features and quality metrics. This stresses the need for transparent reporting and standardized methodologies to promote reproducible and interpretable study findings. ReSurfEMG provides an accessible solution for comprehensive reporting of signal analysis workflows and lays the groundwork for the clinical adoption of state-of-the-art respiratory sEMG methodologies.

## Figures and Tables

**Table 1:** Python packages for EMG analysis.

	BioSPPy	NeuroKit2	Pyemgpipeline	ReSurfEMG
**Filtering**	+	+/−	+	++
**ECG removal**	−	−	−	++
**Envelope extraction**	+	+	+	+
**Event detection**	+	+	−	++
**Waveform** **features**	−	−	−	++
**Quality assessment**	−	+	−	++
**Synthetic data**	+	+	−	++
**Auxiliary physiological signals**	ABP, ACC, BVP, ECG, EDA, EEG, PCG, PPG, resp.	ECG, EDA, EEG. EOG, PPG, resp.	−	Respiratory pressures, flows, volumes

− Not available, +/− Available via external or indirect methods (e.g., third-party integration), + One or more relevant methods implemented, ++ Methods and default settings specifically tailored to respiratory sEMG analysis. Abbreviations: ECG: electrocardiogram; ABP: arterial blood pressure; ACC: accelerometry; BVP: blood volume pulse; EDA: electrodermal activity; EEG: electroencephalogram; EOG: electrooculogram; PPG: photoplethysmogram; resp.: respiration signal.

**Table 2:** ReSurfEMG package.

ReSurfEMG Subpackage	Modules	Description
*data_* *connector*	*config*	Configure default paths for data analysis
	*file_* *discovery*	Detect folders and files
	*converter_* *functions*	Load data in ReSurfEMG standard format
	*tmsisdk_* *lite*	ReSurfEMG adaptation of TMSi SDK library
	*adicht_reader*	ReSurfEMG adaptation for importing .adicht data
	*synthetic_* *data*	Generate synthetic sEMG and auxiliary data
	*data_* *classes*	Object-oriented data processing for TimeSeries data
	*peakset_* *class*	Object-oriented handling of waveform features and quality
*preprocessing*	*filtering*	Remove baseline drift and powerline noise
	*ecg_* *removal*	Remove ECG components
	*envelope*	Calculate sEMG envelope
*postprocessing*	*baseline*	Calculate dynamic sEMG baseline
	*event_* *detection*	Detect breathing events
	*features*	Calculate sEMG and auxiliary data features
	*quality_* *assessment*	Assess sEMG and auxiliary data quality
*helper_* *functions*	*math_* *operations*	Data agnostic math operations
	*visualization*	Visualize basic signal characteristics
	*data_* *classes_quality_assessment*	The quality assessment functions for the data class TimeSeries objects
*pipelines*	*ipy_* *widgets*	Standardized Jupyter Widgets
	*processing*	Standardized signal processing pipelines
	*synthetic_* *data*	Standardized synthetic data generation
*cli*	*cli*	Command line interface: Run the library from the command line outside a Python environment or shell

**Table 3:** Signal processing settings.

Processing Step	Range	Default	Under-Filtering	Over-Filtering
Filtering [18,19,23]				
High-pass cutoff	10–100 Hz	20 Hz	15 Hz	30 Hz
Low-pass cutoff	30–1000 Hz	500 Hz	1000 Hz	500 Hz
Filter order	1–8	3	1	3
ECG removal: Gating [29,30]				
Gate width	0.05–0.40 s	0.20 s	0.15 s	0.30 s
Filling method	Zeros/Prior average/Raw interpolation/RMS interpolation	RMS interpolation	RMS interpolation	RMS interpolation
ECG removal: Wavelet denoising [22]				
Decomposition level	2–6	*floor(log_2_(fs/20))*	N/A	N/A
Fixed threshold	4.0–12.0	4.5	N/A	N/A
Envelope: RMS and ARV [25]				
Window length	0.10–1.00 s	0.25 ms	0.10 ms	0.50 ms
Moving baseline [24,25]				
Window length	1–10 s	7.5 s	7.5 s	7.5 s
Percentile	10th–50th	33rd	20th	33rd

**Table 4:** Calculated sEAdi features and quality aspects with over- and under-filtering and the ReSurfEMG default settings.

Filtering	Under (*n* = 158)	Default (*n* = 158)	Over (*n* = 158)
**Features**			
**Duration (s)**	1.0 (0.2) −12%	1.2 (0.2)	1.4 (0.1) +18%
**T_max_ (s)**	0.5 (0.1) −18%	0.6 (0.1)	0.6 (0.1) +13%
**Amplitude (µV)**	4.0 (0.8) +21%	3.3 (0.7)	1.4 (0.4) −58%
**ETP (µV.s)**	2.1 (0.3) +10%	1.9 (0.4)	1.2 (0.3) −39%
**Pseudo-slope (µV/s)**	12.9 (5.8) +61%	8.0 (1.9)	4.1 (0.9) −49%
**Quality aspects**			
**Pseudo-SNR (.)**	3.1 (0.4) −4%	3.2 (0.5)	1.9 (0.3) −40%
**AUB (%)**	15.6 (3.3) +9%	14.3 (3.7)	20.8 (6.3) +46%
**Bell error (%)**	21.2 (5.0) +73%	12.3 (3.8)	7.1 (2.7) −42%

Numbers are expressed as mean (standard deviation). The % reflects the percentage change relative to parameters calculated using the default filter settings. Abbreviations: Tmax: timing of the peak maximum relative to the waveform start; ETP: electrical time product; SNR: signal-to-noise ratio; AUB: area under the baseline.

## Data Availability

The datasets used and/or analyzed during the current study are available upon reasonable request. The Jupyter notebooks used for analysis, along with synthetic data for testing these notebooks, are publicly available at: Notebooks: https://github.com/resurfemg-org/ReSurfEMG/tree/main/notebooks/researcher_interface/software_paper (accessed on 29 September 2025) and; Synthetic data: https://github.com/resurfemg-org/ReSurfEMG/tree/main/test_data (accessed on 29 September 2025).

## Supplementary Materials

The following supporting information can be downloaded at: https://www.mdpi.com/article/10.3390/s25206465/s1, File S1 AF1_Wavelet_denoising_in_patient_and_simulated_data.pdf. The power spectrum (Pwelch), effects of wavelet denoising order and gating windows on patient and simulated data.

## Abbreviations

The following abbreviations are used in this manuscript:

EMG	Electromyography
sEMG	Surface EMG
ETP	Electrical time product
SNR	Signal-to-noise ratio
AUB	Area under the baseline
ICU	Intensive care unit
ECG	Electrocardiogram
CLI	Command line interface
PyPI	Python Package Index
RMS	Root-mean-square
SWT	Stationary wavelet transform
*n*	Decomposition level
*fc*	Cutoff frequency
*fs*	Sampling frequency
MAD	Median absolute deviation
sEAdi	Surface electrical activity of the diaphragm
ARV	Average rectified value
*cf_hp_*	High-pass cutoff frequency
*cf_lp_*	Low-pass cutoff frequency
CCMO	Central Committee on Research Involving Human Subjects
