# Supplementary material for: ReSurfEMG: A Python Package for Comprehensive Analysis of Respiratory Surface EMG
Source: Sensors (Basel). 2025 Oct 19;25(20):6465. doi: 10.3390/s25206465 (PMC12567628; doi:10.3390/s25206465)
Supplement: Supplementary file 1 [file sensors-25-06465-s001.zip › sensors-3899039-supplementary.pdf]

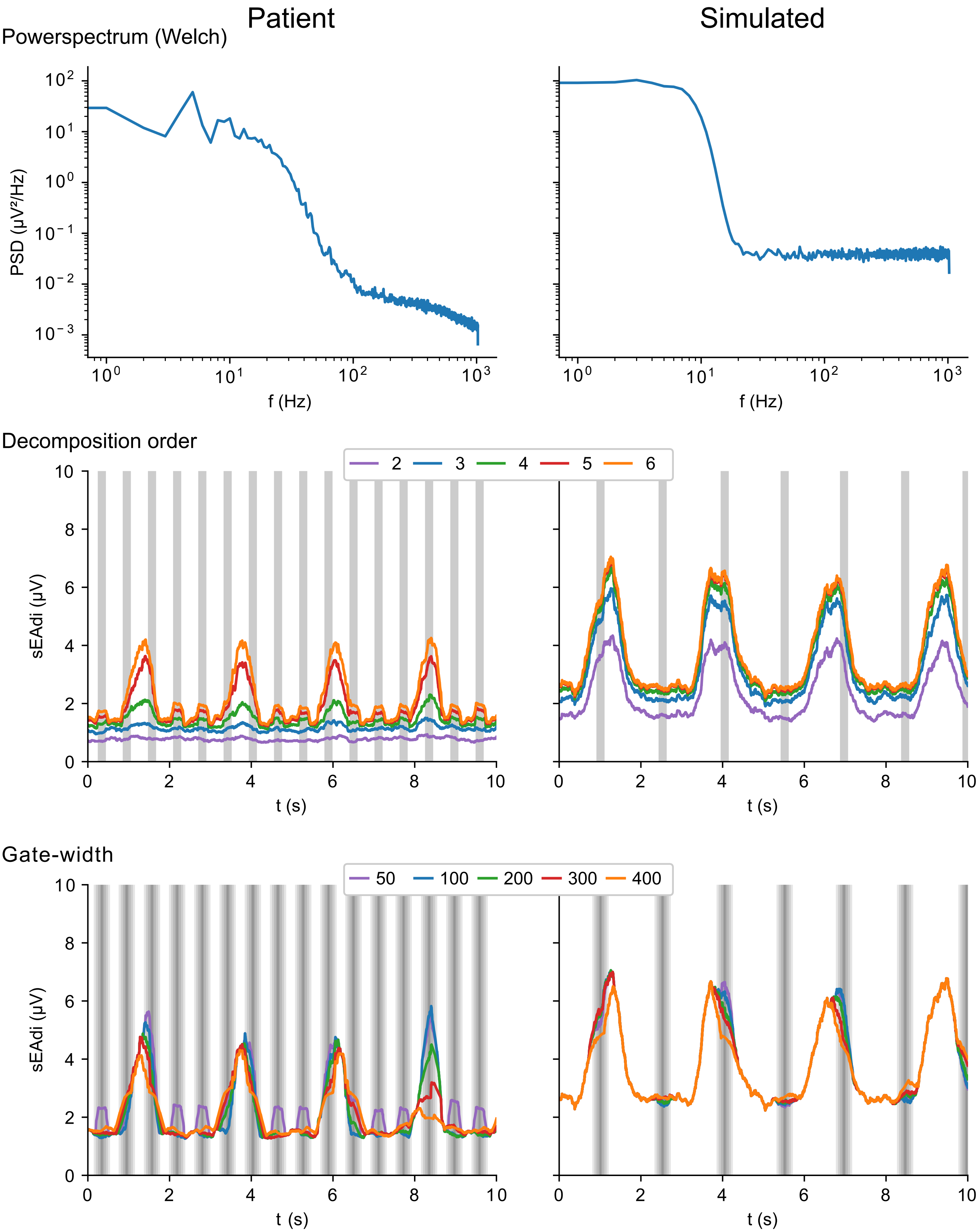

Figure 1 - The Welch power spectrum (top), effects of wavelet denoising order (mid) and gate windows (bottom) on patient (left) and simulated data (right). Simulated sEMG data had a stable frequency content for frequencies between 200 and 2048 Hz, whereas the patient power spectrum continues to fall to approximately 10^{-3} μV²/Hz. As a result, wavelet denoising still shows a reasonable sEAdi waveform for decomposition order 2 in the simulated data, whereas the waveform in the patient data is near-flat. These differences illustrate that synthetic signals, generated by modulating white noise with a cyclic reference, do not replicate the spectral characteristics of physiological EMG. Therefore, their application is intended for code testing and demonstration purposes. The gating results are shown as reference for the absolute sEAdi amplitude.
